# A nuclear targeted dual-photosensitizer for drug-resistant cancer therapy with NIR activated multiple ROS†

**DOI:** 10.1039/c6sc00737f

**Published:** 2016-03-11

**Authors:** Zhengze Yu, Wei Pan, Na Li, Bo Tang

**Affiliations:** a College of Chemistry, Chemical Engineering and Materials Science, Collaborative Innovation Center of Functionalized Probes for Chemical Imaging in Universities of Shandong, Key Laboratory of Molecular and Nano Probes, Ministry of Education, Shandong Provincial Key Laboratory of Clean Production of Fine Chemicals, Shandong Normal University Jinan 250014 P. R. China tangb@sdnu.edu.cn

## Abstract

Photodynamic therapy against cancer, especially multidrug resistant cancer, is limited seriously due to the efflux of photosensitizer molecules by P-glycoprotein, which leads to insufficient production of reactive oxygen species (ROS). For the purpose of abundant ROS generation and effective therapeutic response, herein, we firstly design and fabricate a nuclear targeted dual-photosensitizer for photodynamic therapy against multidrug resistant cancer. Molecule-photosensitizer Ce6 was selected and modified on the surface of core/shell structure nano-photosensitizer upconversion@TiO_2_ and then nuclear targeted peptides TAT were anchored for nuclear targeting. Through selective doping of rare earth elements Er and Tm, multiple ROS (˙OH, O_2_˙^−^, and ^1^O_2_) can be generated for the dual-photosensitizer and realize their functions synergistically using a single 980 nm NIR excitation. The nano-sized photosensitizer accompanied with nuclear targeting can effectively generate multiple ROS in the nucleus regardless of P-glycoprotein and directly break DNA double strands, which is considered as the most direct and serious lesion type for cytotoxic effects. Therefore, enhanced photodynamic therapy can be achieved against multidrug resistant cancer. *In vitro* and *in vivo* studies confirmed the excellent therapeutic effect of the dual-photosensitizer against cancer cells and drug-resistant cancer cells, as well as xenograft tumor models.

## Abstract 2

A nuclear targeted dual-photosensitizer was developed for photodynamic therapy against multidrug resistant cancer. Multiple reactive oxygen species (ROS) could be generated in the nucleus to directly break DNA double strands with a single 980 nm NIR laser irradiation, regardless of drug resistance.

## Graphical abstract

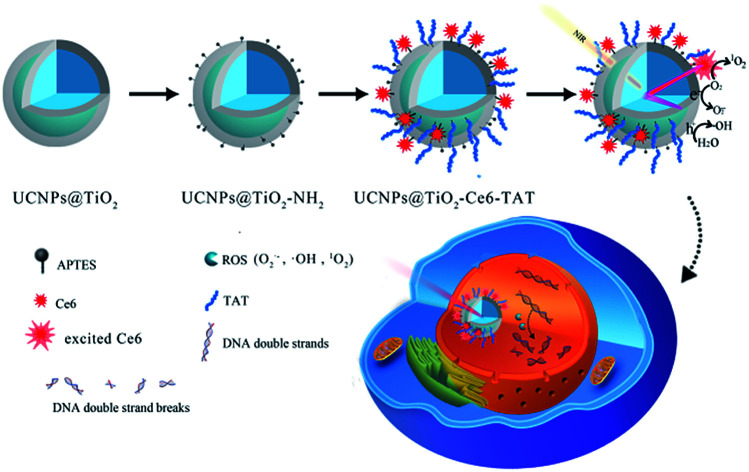

## Introduction

Cancer is undeniably one of the most intricate and refractory diseases with increasing morbidity in recent years.^1^ The extremely high mortality makes it a serious threat to human health.^2,3^ Photodynamic therapy (PDT), as an emerging therapeutic modality, has undergone many investigations and plays a key role in current cancer therapy.^4–7^ However, the clinic application of PDT is severely limited against multidrug resistant (MDR) cancer.^8,9^ The overexpression of P-glycoprotein (P-gp) transporters on the cell membrane is the primary cause of MDR, which functions as an ATP-dependent efflux pump responsible for the unidirectional expelling of molecules across the cell membrane.^10,11^ The efflux of traditional photosensitizer molecules means the intracellular photosensitizer concentration fails to reach the lethal threshold, which further leads to insufficient ROS generation and an inefficient therapeutic response.^10,11^ For the purpose of abundant ROS generation against MDR cancer, a dual-photosensitizer is superior for multiple ROS generation because the dual-photosensitizer model can combine the advantages of molecule-photosensitizers and nano-photosensitizers, which can amplify the therapeutic effects and its suitable size and scale contribute to exceed the limit of the P-gp efflux channel.^12–15^ However, the ultraviolet excitation of nano-photosensitizers and the visible excitation of molecule-photosensitizers have poor tissue penetration.^16–19^ Besides, two different excitations make its application inconvenient. Upconversion nanoparticles can achieve multiple emission through doping various rare earth ion sensitizers^20–25^ and the NIR excitation meets the needs of deep tissue applications,^26,27^ so they are ideal candidates for dual-photosensitizers.

Moreover, the inherent nature of ROS, with a short life and diffusion distance, is another drawback of traditional PDT.^28–30^ As is well known, the nucleus contains most of the intracellular genetic materials, directs their functions and has a prominent role in cell proliferation and differentiation.^31–34^ Therefore, it is the final destination of many widely used chemotherapy drugs in clinics, such as doxorubicin (Dox), and cisplatin (CDDP), which realize their therapeutic function by inserting in or coupling to the DNA double strands to prevent DNA replication.^35^ Considering that DNA double strand breaks are the most direct and serious lesion type for cytotoxicity and that ROS can afford this *via* oxidative damage,^36,37^ nuclear targeted generation of multiple ROS can greatly improve the therapeutic effects, because their nuclear targeting ability can make the ROS directly function at the correct place. Thus, it is highly desirable to develop a nuclear-targeted nanoagent which could generate multiple ROS under a NIR laser against drug-resistant cancer.

**Scheme 1:**
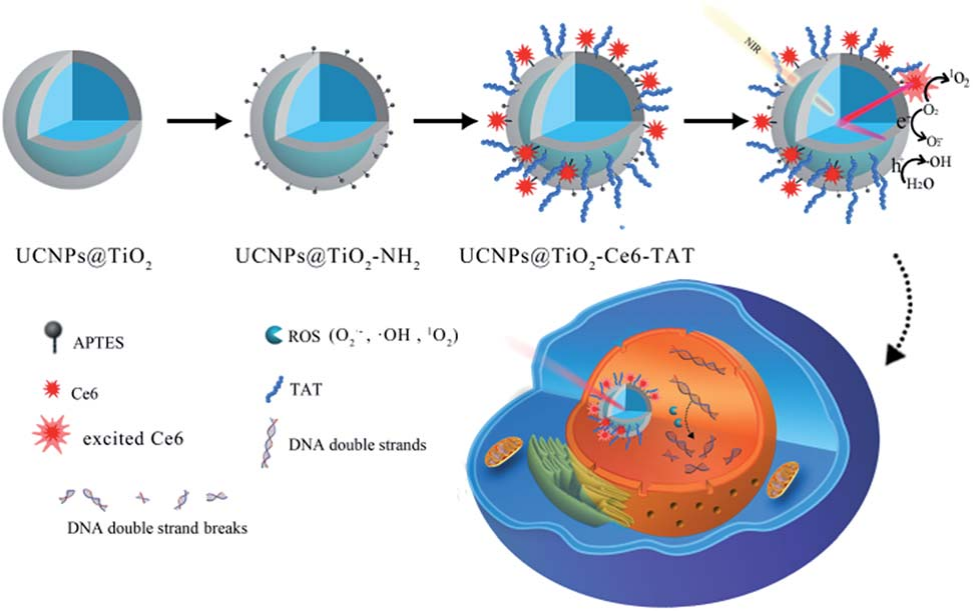
Schematic illustration of the synthetic process of the nuclear targeted dual-photosensitizer UCNPs@TiO_2_-Ce6-TAT, multiple ROS generation under a single 980 nm NIR laser excitation and inducing DNA double strand breaks.

Herein, we design and fabricate a novel nuclear targeted dual-photosensitizer for PDT, NaFY_4_:Yb,Er,Tm@TiO_2_-Chlorin e6-TAT (abbreviated as UCNPs@TiO_2_-Ce6-TAT). For the first time, we combined a nano-photosensitizer and molecule-photosensitizer together to generate multiple ROS with one NIR excitation wavelength. The molecule-photosensitizer Ce6 was selected due to its fluorescence spectrum match and modified on the surface of the core/shell structure nano-photosensitizer UCNPs@TiO_2_ and then nuclear targeted peptides TAT were anchored for the nuclear penetration purpose. The UCNPs were designed to be excited with a 980 nm NIR laser and emit in the ultraviolet and visible region by doping with lanthanides Tm and Er. Subsequently, the emission at 362 nm and 655 nm of the UCNPs can be absorbed by the TiO_2_ layer and Ce6 molecules, respectively *via* fluorescence resonance energy transfer (FRET) to generate a variety of ROS, including ˙OH, O_2_˙^−^, and ^1^O_2_. On this occasion, simultaneous generation of multiple ROS can be achieved with a single 980 nm NIR excitation. The NIR light irradiation allows deeper penetration and lower risk of normal tissue damage. TAT peptides were employed to translocate the nanoparticles into the nuclear region and made the ROS accumulate in the nucleus. The accumulation of large amounts of ROS in the cell nucleus can break DNA double strands and further lead to cell death. Therefore, this dual-photosensitizer can realize its therapeutic function synergistically and have a better therapeutic effect. The specific structure and design of the dual-photosensitizer are depicted in Scheme 1.

## Results and discussion

### Synthesis and characterization of the nuclear targeted dual-photosensitizer

**Fig. 1:**
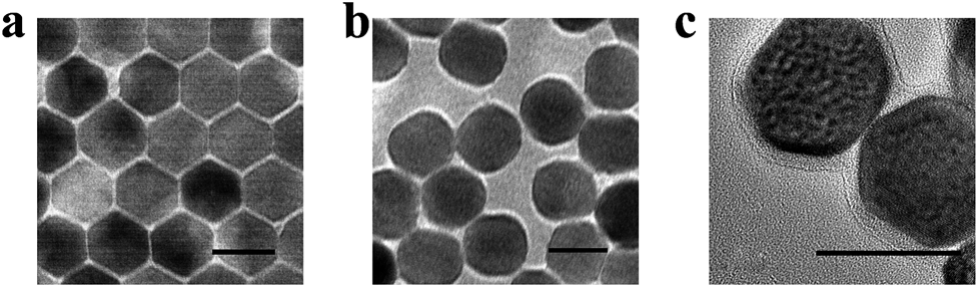
High resolution transmission electron microscopy (HRTEM) images of OA coated NaYF_4_:Yb^3+^,Er^3+^,Tm^3+^ (a); OA free NaYF_4_:Yb^3+^,Er^3+^,Tm^3+^ (b); NaYF_4_:Yb^3+^,Er^3+^,Tm^3+^@TiO_2_ (c). Scale bars are 25 nm.

**Fig. 2:**
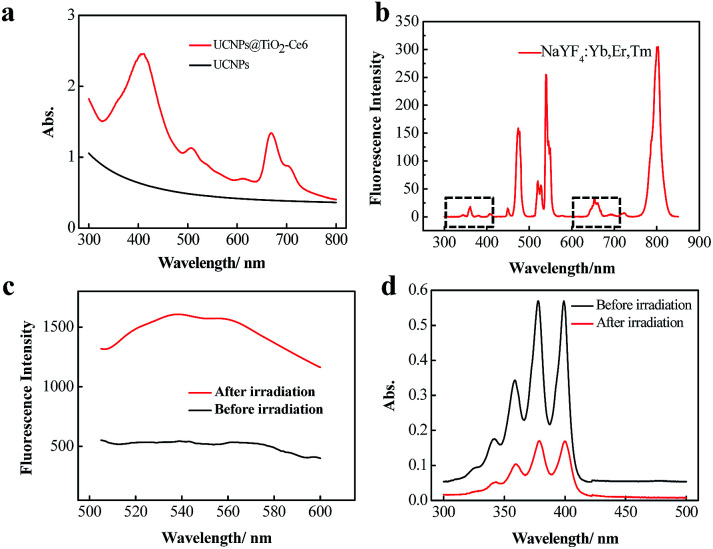
Characterization of the FRET and multiple ROS generation. (a) Absorption spectra of the pure UCNPs and UCNPs@TiO_2_-Ce6. (b) The fluorescence emission spectrum of NaYF_4_:Yb^3+^,Er^3+^,Tm^3+^. The two obvious absorption regions around 362 nm and 655 nm were due to Tm and Er doping, respectively. The fluorescence emission spectra of DBZTC (*λ*_ex_ = 476 nm, *λ*_em_ = 505–600 nm) (c) and absorption spectra of ABMD (d) before and after treatment with UCNPs@TiO_2_-Ce6 and 980 nm laser irradiation.

Heterogeneous core–shell nanoparticles with a core of β-phase upconversion NaFY_4_ co-doped with 20% Yb^3+^, 0.2% Tm^3+^, 0.1% Er^3+^ (NaFY_4_:Yb^3+^,Tm^3+^,Er^3+^) and a shell layer of TiO_2_ were synthesized through a solvothermal method and subsequent epitaxial growth of TiO_2_.^38,39^Fig. 1a–c display the high resolution transmission electron microscopy (HRTEM) images of oleic acid (OA)-conjugated UCNPs, OA free UCNPs and UCNPs@TiO_2_. OA-UCNPs in cyclohexane had uniform hexagonal morphology (β-phase) with sizes of about 25 nm. OA free UCNPs were obtained after hydrochloric acid treatment, and exhibited the same size and good monodispersity in the aqueous phase. Subsequently, the TiO_2_ layer was modified on the UCNPs and can be observed clearly with a homogeneous thickness about 3 nm (Fig. 1c). After functionalized with amino groups, UCNPs@TiO_2_-Ce6 was obtained by coupling the carboxyl groups of the Ce6 molecules and the amino groups on the surface. As shown in the UV-vis spectrum (Fig. 2a), two obvious absorption regions of UCNPs@TiO_2_-Ce6 in the UV and around the 650 nm region appeared, corresponding to the absorbance of TiO_2_ and Ce6, which confirmed the successful modification of the Ce6 molecules. Finally, TAT peptides were also coupled on the surface by forming amido bonds. The zeta potentials of each step provided further evidence for the fabrication process, *i.e.* +3.5 ± 0.4 mV, −27.5 ± 0.7 mV, +24.6 ± 0.6 mV and +6.0 ± 0.3 mV. The content of Ce6 was calculated to be 0.88 μmol mg^−1^ UCNPs@TiO_2_ according to a standard linear calibration curve (Fig. S1, ESI†) and the content of TAT peptide was 5.1 μmol mg^−1^ UCNPs@TiO_2_ using a nanodrop-based method.

### Multiple ROS generation

We first assessed whether the dual-photosensitizer could generate multiple ROS through 980 nm NIR laser irradiation. The fluorescence spectrum of NaYF_4_:Yb^3+^,Tm^3+^,Er^3+^ is given in Fig. 2b. The main spectral emission peaks corresponded to the transformations: ^1^D_2_–^3^H_6_ (362 nm), ^1^G_4_–^3^H_6_ (476 nm), and ^3^H_4_–^3^H_6_ (800 nm) of Tm^3+^, and ^2^H_11/2_–^4^H_15/2_ (521 nm), ^4^S_3/2_–^4^S_15/2_ (541 nm), and ^4^F_19/2_–^4^I_15/2_ (655 nm) of Er^3+^. Combined with the spectrum of UCNPs@TiO_2_-Ce6, we found that the absorption regions of TiO_2_ and Ce6 overlapped well with the emission peaks at 362 nm and 655 nm which enabled the activation of TiO_2_ and Ce6 to generate multiple ROS. Then 2-chloro-1,3-dibenzothiazolinecyclohexene (DBZTC)^40^ and 9,10-anthracenediyl-bis(methylene)dimalonic acid (ABMD)^41^ were employed to detect the generated O_2_˙^−^ and ^1^O_2_ under 980 nm laser irradiation. As shown in Fig. 2c and d, an obvious fluorescence intensity increase of DBZTC and an absorption decrease of ABMD were observed, which verified the generation of multiple ROS. These results indicated that UCNPs@TiO_2_-Ce6 could efficiently generate multiple ROS under 980 nm NIR laser irradiation.

### Colocalization and intracellular tracking profile

**Fig. 3:**
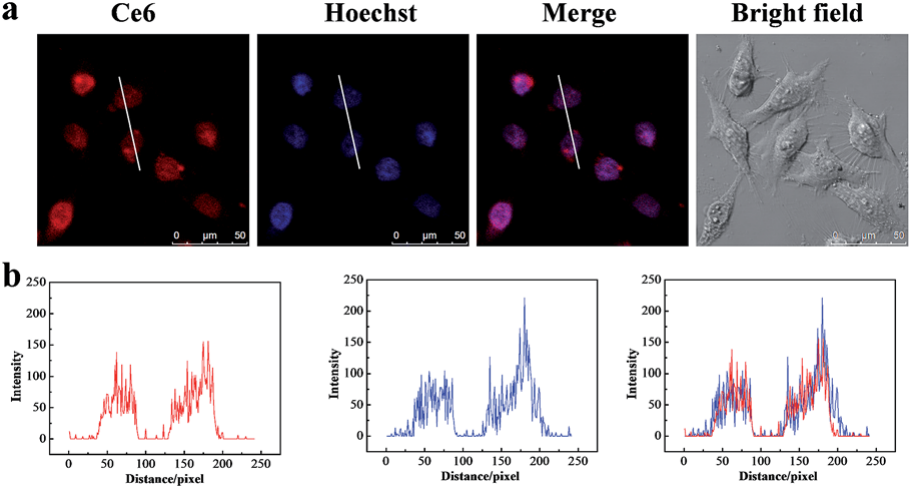
Nuclear targeting assay of MCF-7 cells. (a) Colocalization images of the nuclear targeted dual-photosensitizer and the nuclei using confocal laser scanning microscopy (CLSM). MCF-7 cells were incubated with UCNPs@TiO_2_-Ce6-TAT for 12 h before measurement. Confocal images of the nuclear targeted dual-photosensitizer (*λ*_ex_ = 633 nm, *λ*_em_ = 650–700 nm), Hoechst 33342 stained nuclei (*λ*_ex_ = 405 nm, *λ*_em_ = 430–480 nm) and the overlay channel. (b) The quantification of fluorescence intensity of the line scanning profiles of the corresponding confocal images in (a).

**Fig. 4:**
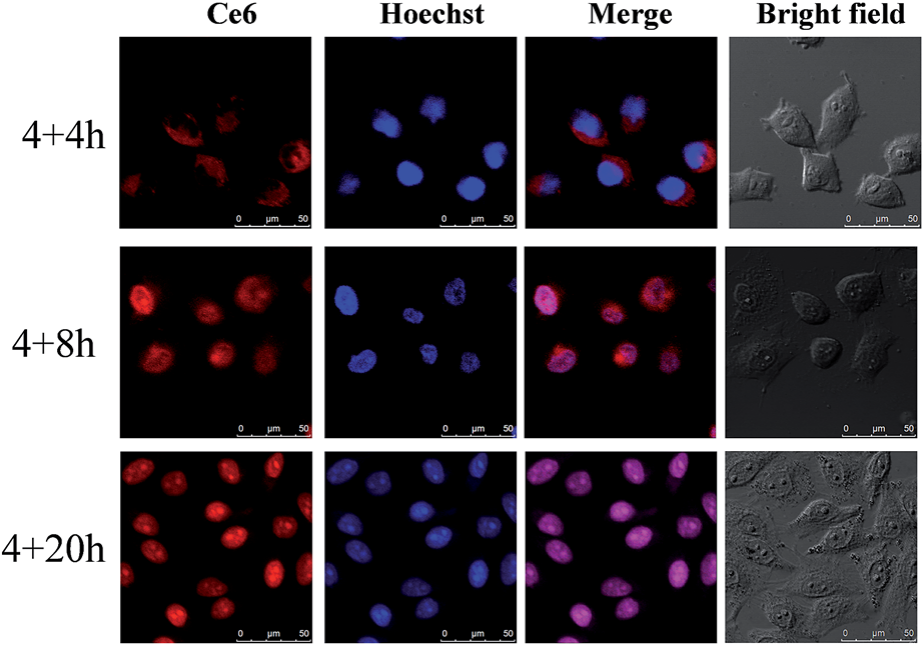
Intracellular tracking of the nuclear targeted dual-photosensitizer. Confocal images of MCF-7 after incubation with UCNPs@TiO_2_-Ce6-TAT for different times. MCF-7 cells were first incubated with UCNPs@TiO_2_-Ce6-TAT (0.1 mg mL^−1^) for 4 h. The excess nanoparticles were then removed and the cells were incubated with fresh culture media for an additional 4, 8, and 20 h. Confocal images of Ce6 (*λ*_ex_ = 633 nm, *λ*_em_ = 650–700 nm), Hoechst 33342 stained nuclei (*λ*_ex_ = 405 nm, *λ*_em_ = 430–480 nm), and the overlay channel.

To assess whether the TAT peptide-modified dual-photosensitizer has the capability of targeting the nucleus, the intracellular distribution of the designed dual-photosensitizer was investigated using confocal laser scanning microscopy (CLSM). A nuclear targeted dye, Hoechst 33342 was employed to label the nuclei, and has a bright blue fluorescence when combined with DNA double strands. The fluorescence of Ce6 was employed to label the TAT peptide-modified dual-photosensitizer. As shown in Fig. 3a, the fluorescence of Hoechst 33342 and Ce6 overlapped well and an obvious purple signal was observed in the merged image of MCF-7 cells. The same result was obtained from the fluorescence intensity quantification of the line scanning profiles (Fig. 3b). Bio-TEM images of MCF-7 cells provided further evidence for the nuclear targeting of the TAT peptide-modified dual-photosensitizer and most of the dual-photosensitizers modified with TAT peptides were located inside the nucleus (Fig. S2, ESI†). A nuclear targeting assay was also carried out in doxorubicin-resistant human breast cancer cells (MCF-7/Dox) which overexpress *p*-glycoprotein and the confocal images showed the excellent nuclear targeting ability of this dual photosensitizer in drug-resistant cancer cells as well (Fig. S3, ESI†). For comparison purposes, nanoparticles UCNPs@TiO_2_-Ce6 without TAT peptides were employed to confirm the function of the TAT peptides. The results showed that most of nanoparticles stayed in the cytoplasm after incubation (Fig. S4, ESI†) which suggested that the TAT peptides played a crucial role in the nuclear targeting of the dual-photosensitizer. These results indicated that UCNPs@TiO_2_-Ce6-TAT achieved the targeting of nuclear localization. The intracellular tracking of UCNPs@TiO_2_-Ce6-TAT for different incubation times (8 h, 12 h, and 24 h) was also investigated. As shown in Fig. 4, only a small overlay area appeared on the edge of the nuclei when the incubation time was 8 h, demonstrating that most of UCNPs@TiO_2_-Ce6-TAT still remained in the cytoplasm and was prepared to enter the nuclei. After another 4 h of incubation, most of the dual-photosensitizer was located inside the nuclei and only a small part was in the cytoplasm near the nuclei. Notably, there was very strong red fluorescence in the nuclear region while there was a very slight red fluorescence in the cytoplasm, suggesting that almost all of the dual-photosensitizer was in the nuclei when the incubation time reached 24 h.

### The therapeutic effect of the nuclear targeted dual-photosensitizer in living cells

**Fig. 5:**
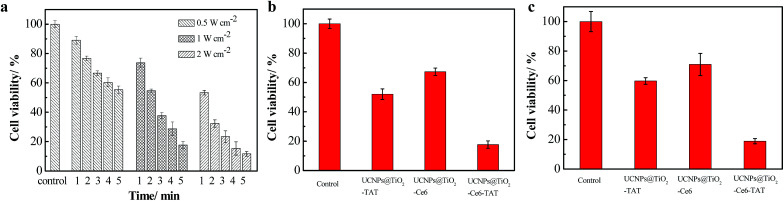
*In vitro* MTT assay. (a) Cell viability of MCF-7 cells incubated with UCNPs@TiO_2_-Ce6-TAT (0.1 mg mL^−1^) under various parameters (irradiation time and power). Cell viability was measured 24 h after irradiation. Cell viability of MCF-7 cells (b) and MCF-7/Dox cells (c) were also measured after incubation with UCNPs@TiO_2_-TAT, UCNPs@TiO_2_-Ce6, and UCNPs@TiO_2_-Ce6-TAT or without treatment under 980 nm using 1 W cm^−2^ laser irradiation for 5 min.

To verify the therapeutic efficiency of the nuclear targeted dual-photosensitizer UCNPs@TiO_2_-Ce6-TAT, we assessed the cytotoxicity using an *in vitro* MTT assay. Firstly, we explored the parameters of the NIR irradiation (irradiation power and time). After incubation with UCNPs@TiO_2_-Ce6-TAT for 12 h, the MCF-7 cells were treated with different laser powers (0.5, 1, and 2 W cm^−2^) and different irradiation times (1, 2, 3, 4, and 5 min). As can be seen in Fig. 5a, the cell viability decreased with the increase in laser power and irradiation time. Considering the therapeutic effects and the lower phototoxicity to normal cells, the parameters of 1 W cm^−2^ and 5 min were chosen in the following therapeutic application. To reveal the pivotal role of the nuclear targeted peptides and enhanced effect of the multiple ROS generation, three groups of MCF-7 cells were treated with UCNPs@TiO_2_-TAT, UCNPs@TiO_2_-Ce6, and UCNPs@TiO_2_-Ce6-TAT for 12 h, respectively, and then irradiated with a 980 nm NIR laser. As presented in Fig. 5b, nanoparticles without Ce6 or TAT treatment showed relatively high cell viability (52.0% and 67.3%). Importantly, the viability of cells treated with UCNPs@TiO_2_-TAT was lower than those treated with UCNPs@TiO_2_-Ce6, which was mainly due to the inherent nature of the ROS *i.e.*, a short life and limited diffusion distance. Noticeably, only 17.7% cells were still alive when incubated with UCNPs@TiO_2_-Ce6-TAT and irradiated with the NIR laser, which demonstrated its superiority as a novel combined therapeutic agent.

We then assessed whether the nuclear targeted dual-photosensitizer had an effect on drug-resistant cancer cells. As can be seen in Fig. 5c, only 15.9% of cells survived after treatment with UCNPs@TiO_2_-Ce6-TAT while the viability of the cells in comparative groups was more than 50%. These MTT results indicated that the nuclear targeted dual-photosensitizer had an excellent therapeutic effect not only for MCF-7 cells but also for MCF-7/Dox cells. As a control, the viability of cells treated with only UCNPs@TiO_2_-Ce6-TAT or only 980 nm NIR laser irradiation were also investigated. The results showed that more than 90% of cells were still alive in both samples, indicating that UCNPs@TiO_2_-Ce6-TAT possessed good biocompatibility and that the irradiation conditions we chose had negligible side effects (Fig. S5, ESI†).

**Fig. 6:**
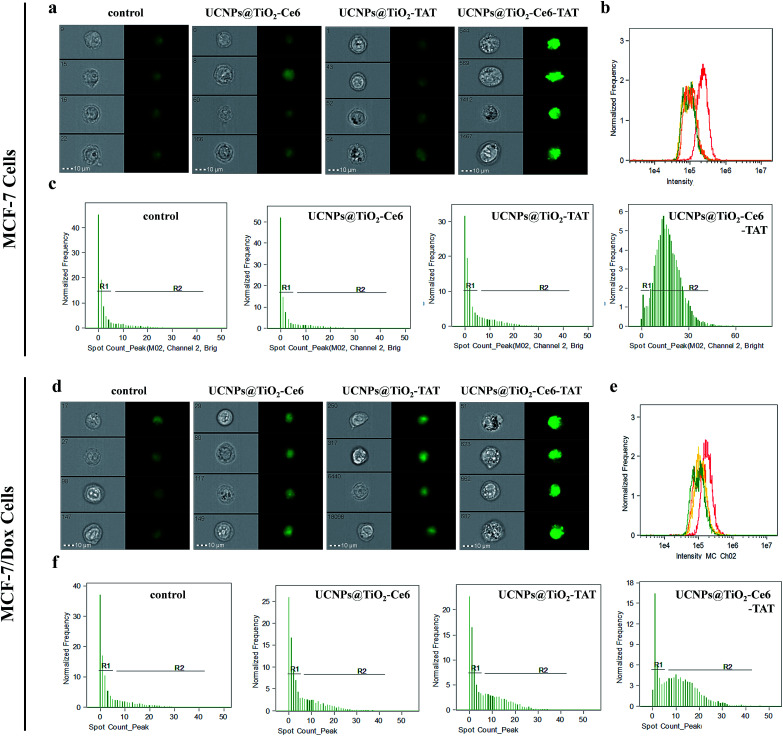
*In vitro* verification of DNA double strand breaks in MCF-7 cells (top) and MCF-7/Dox cells (bottom) *via* γ-H2AX immunofluorescence staining using imaging flow cytometry. (a and d) Cell images after different treatments (*λ*_ex_ = 488 nm, *λ*_em_ = 500–560 nm). Two kinds of cells were incubated with UCNPs@TiO_2_-TAT, UCNPs@TiO_2_-Ce6, and UCNPs@TiO_2_-Ce6-TAT or without treatment, followed by 5 min of irradiation. (b and e) Flow cytometry data of the fluorescence intensity of cells under different treatments corresponding to the cells in (a) and (d). (c and f) The distribution of cells according to the number of γ-H2AX foci per cell.

To verify our hypothesis that the multiple ROS induced DNA double strand breaks, DNA immunofluorescence staining (γ-H2AX staining) studies were carried out using imaging flow cytometry (IFC) to make a detailed statistical analysis. DNA damage, *i.e.* double-strand breaks will induce phosphorylation of the variant histone of the H2A protein family, H2AX. This newly phosphorylated protein γ-H2AX is responsible for recruitment and localization of the DNA repair mechanism and it is also an efficient biomarker for the detection of double strand breaks.^42–44^Fig. 6a exhibits the MCF-7 cell images of different treatments. As can be seen, the green fluorescence signal of cells treated with UCNPs@TiO_2_-Ce6-TAT was much brighter than the cells treated with UCNPs@TiO_2_-TAT, UCNPs@TiO_2_-Ce6, or without treatment. The statistical data of the cell fluorescence intensity were consistent with the above results (Fig. 6b and Table 1), which indicated that UCNPs@TiO_2_-Ce6-TAT can cause much severe DNA damage. Moreover, it was found MCF-7 cells treated with UCNPs@TiO_2_-Ce6-TAT showed obvious γ-H2AX foci (shown as greet fluorescent spots) and the cells with relatively more γ-H2AX foci possessed a high percentage (Fig. 6c). The number of γ-H2AX foci per cell was calculated to be enhanced by more than 4 times for the cells treated with UCNPs@TiO_2_-Ce6-TAT compared to cells with other controls (Table 1a). We also conducted this immunofluorescence staining assay in the MCF-7/Dox cells to evaluate the degree of DNA damage. Similarly, the data showed that the MCF-7/Dox cells treated with UCNPs@TiO_2_-Ce6-TAT exhibited the strongest fluorescence signal, the highest mean fluorescence intensity and the maximum value of γ-H2AX foci number (Fig. 6d–f, and Table 1b). These results confirmed that the designed nuclear targeted dual-photosensitizer was capable of causing DNA double strand breaks most severely as expected and had the best therapeutic effect both in MCF-7 cells and drug-resistant MCF-7/Dox cells.

Statistical data of the mean fluorescence intensity, percentage of *R*1 and *R*2 regions, and mean number of γ-H2AX foci per cell in MCF-7 cells (a) and MCF-7/Dox cells (b)

**Table:** 

(a)				
Samples (MCF-7 cells)	Mean fluorescence intensity	*R*1%	*R*2%	Mean number of γ-H2AX foci per cell
Control	100 774	82.7	15.5	2.19
UCNPs@TiO_2_-Ce6	122 050	82.8	15.4	4.17
UCNPs@TiO_2_-TAT	131 133	74.6	22.6	4.93
UCNPs@TiO_2_-Ce6-TAT	240 334	6.5	90.9	16.36

**Table:** 

(b)				
Samples (MCF-7/Dox cells)	Mean fluorescence intensity	*R*1%	*R*2%	Mean number of γ-H2AX foci per cell
Control	101 701	75.9	21.8	3.77
UCNPs@TiO_2_-Ce6	123 910	66.3	30.7	4.28
UCNPs@TiO_2_-TAT	135 413	59.7	37.4	5.15
UCNPs@TiO_2_-Ce6-TAT	183 963	25.5	70.7	11.16

### *In vivo* application

**Fig. 7:**
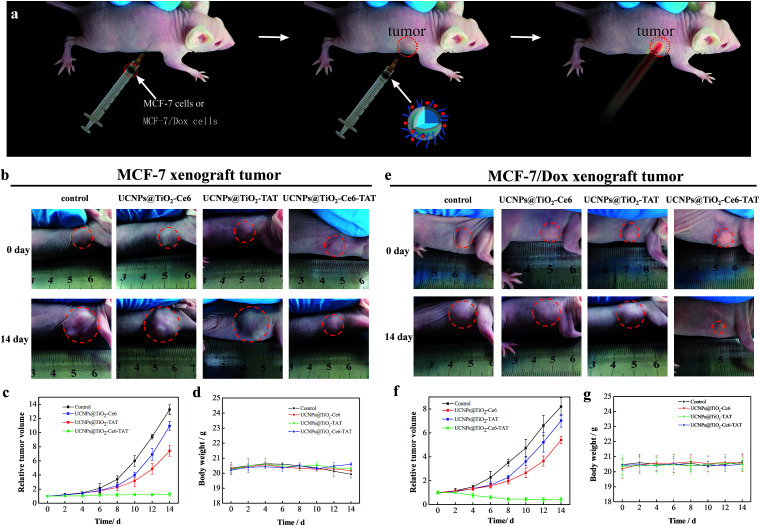
*In vivo* application of the nuclear targeted dual-photosensitizer in a mouse model with xenograft MCF-7 tumor (left) or MCF-7/Dox tumor (right). (a) Schematic illustration of the *in vivo* therapeutic process. (b and e) Photographs of the mice taken before treatment (0 days) and at 14 days with different treatments: control, UCNPs@TiO_2_-Ce6, UCNPs@TiO_2_-TAT, and UCNPs@TiO_2_-Ce6-TAT with irradiation for 5 min in all groups. A dosage of nanoparticles in PBS (1 mg mL^−1^, 50 μL) was administrated intratumorally for all groups of mice (*n* ≥ 5). Tumor growth curves (c and f) and mice body weight curves (d and g) of different groups of tumor-bearing mice. They were measured at 2 day intervals for 14 days. The power of irradiation was 1 W cm^−2^.

**Fig. 8:**
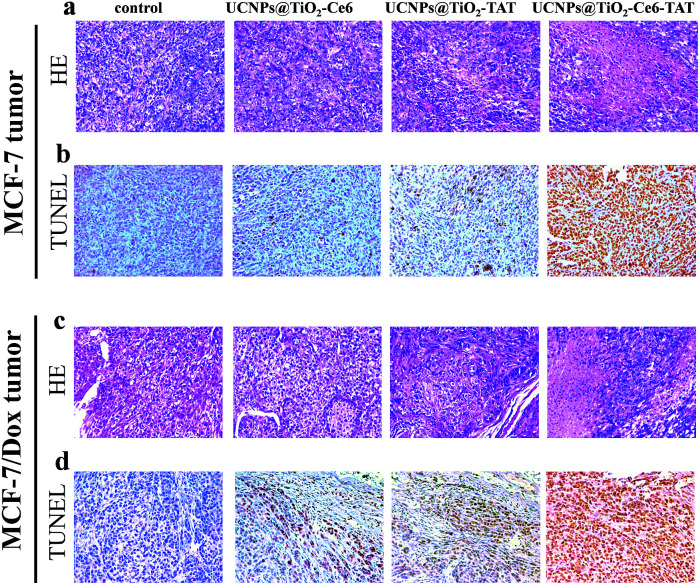
H&E staining and TUNEL staining of MCF-7 tumor (top) and MCF-7/Dox tumor (bottom) slides. The tumors were treated differently: control group, UCNPs@TiO_2_-Ce6, UCNPs@TiO_2_-TAT, and UCNPs@TiO_2_-Ce6-TAT with irradiation for 5 min. The power of irradiation was 1 W cm^−2^.

Based on the excellent therapeutic effect *in vitro*, the nuclear targeted dual-photosensitizer was expected to have great potential as a novel nanoagent for tumor therapy *in vivo*. We then investigated its ability against cancer in a mouse model. Xenograft mouse models (the mice were treated with MCF-7 cells and MCF-7/Dox cells, respectively) were then employed to evaluate the therapeutic effect of UCNPs@TiO_2_-Ce6-TAT in tumor tissue. The brief xenograft and therapeutic process are illustrated in a schematic diagram (Fig. 7a). MCF-7 cells and MCF-7/Dox cells (1 × 10^6^ cells per mouse) were first injected into the flank of the mice. After the formation of a solid tumor (about 150 mm^3^), UCNPs@TiO_2_-TAT, UCNPs@TiO_2_-Ce6, and UCNPs@TiO_2_-Ce6-TAT (1 mg mL^−1^, 50 μL) were then directly injected into the solid tumors of the mice, respectively and the tumor region underwent 980 nm NIR laser irradiation of 1 W cm^−2^ for 5 min. The change in the tumor volume was measured over a period of 14 days without extra injection and laser irradiation. As can be seen in Fig. 7b and c, after 14 days the tumor volume of the MCF-7 xenograft tumor stayed the same as at day 0 when treated with UCNPs@TiO_2_-Ce6-TAT upon NIR laser irradiation for 5 min, and the MCF-7/Dox xenograft tumor volume reduced to almost one-third of its original size (Fig. 7e and f). However, in the control group of mice treated with PBS buffer, the tumor size was found to increase about 13-fold and 8-fold over this period for the MCF-7 and MCF-7/Dox xenograft tumors, respectively (black line in Fig. 7c and f). Two kinds of tumors were also investigated with UCNPs@TiO_2_-TAT and UCNPs@TiO_2_-Ce6 for comparison. The changes in the tumor volume were monitored in the same way. As shown in the photographs and the curves of tumor volume, the tumors boosted rapidly for each sample (Fig. 7c and f). The differences in tumor volume of these groups indicated that the UCNPs@TiO_2_-Ce6-TAT had an excellent therapeutic effect compared to the others for MCF-7 tumors, as well as for MCF-7/Dox tumors. Body weight is an essential parameter to evaluate whether the material or treatment methods had systemic toxicity to the body. As shown in Fig. 7d and g, the body weight of all groups remained almost unchanged over 14 days, implying that the treatments avoided unpleasant side effects successfully. The antitumor mechanisms were further analyzed by histological examination. As given by terminal deoxynucleotidyl transferase dUTP nick end labeling (TUNEL) staining images in Fig. 8, both MCF-7 and MCF-7/Dox xenograft tumors treated with UCNPs@TiO_2_-Ce6-TAT had extensive regions of apoptotic cells, while there were only few apoptosis cells present in the tumors treated with UCNPs@TiO_2_-TAT, UCNPs@TiO_2_-Ce6, and the control. The hematoxylin/eosin (H&E) staining images also exhibited a large area of nuclear shrinkage and fragmentation only in the tumor group treated with UCNPs@TiO_2_-Ce6-TAT, which was consistent with the results of TUNEL staining. The histological effect of UCNPs@TiO_2_-Ce6-TAT on the major organs (liver, lung, spleen, kidney, and heart) of healthy mice was tested at 7 days after intratumor injection and no histopathological abnormalities were observed in all these organs (Fig. S6, ESI†). These results indicated that the nuclear targeted dual-photosensitizer is highly effective against cancer cells and drug-resistant cancer cells and has no side effects to normal tissues.

## Conclusions

In conclusion, we have presented a novel nuclear targeted dual-photosensitizer for PDT against MDR cancer by combining a core/shell structure nano-photosensitizer UCNPs@TiO_2_, a molecule-photosensitizer Ce6 and nuclear targeted peptides TAT. By selective doping of Er^3+^ and Tm^3+^, this dual-photosensitizer allows the generation of multiple ROS (˙OH, O_2_˙^−^, and ^1^O_2_) through a single 980 nm NIR excitation *via* FRET and realizes the therapeutic function synergistically. The nanosized dual-photosensitizers maintained the intracellular concentration of the photosensitizer and generated ROS regardless of whether the cell was drug resistant or not. Moreover, considering the key role of the nucleus in the cell and a better therapeutic response, we conjugated TAT peptides on the surface to break through the drug resistance and locate the nuclear targeted dual-photosensitizer in the nucleus. Thus, the generated ROS can efficiently destroy the function of the nucleus through breaking the DNA double strands, finally leading to cell death. MTT assay indicated that the designed nuclear targeted dual-photosensitizer is superior to the controls and it also reveals that the nuclear targeting plays a pivotal role in inducing cell death. Imaging flow cytometry data confirmed that the breaks of DNA double strands can be achieved as expected in living cells. *In vivo* studies demonstrated that the tumor can be dramatically reduced owing to the excellent therapeutic effect of the designed nuclear targeted dual-photosensitizer. We anticipate that this novel strategy has great potential application for MDR cancer therapy.

## Conflict of interest

The authors declare no competing financial interests.

## Supplementary Material

SC-007-C6SC00737F-s001
