# Supplementary material for: A nuclear targeted dual-photosensitizer for drug-resistant cancer therapy with NIR activated multiple ROS
Source: Chem Sci. 2016 Mar 11;7(7):4237–44. doi: 10.1039/c6sc00737f (PMC6013803; doi:10.1039/c6sc00737f)
Supplement: SC-007-C6SC00737F-s001 [file SC-007-C6SC00737F-s001.pdf]

Electronic Supplementary Material (ESI) for Chemical Science.
This journal is © The Royal Society of Chemistry 2016

Supplementary information

A nuclear targeted dual-photosensitizer for drug-resistant cancer therapy with NIR activated multiple ROS

Zhengze Yu, Wei Pan, Na Li* and Bo Tang*

College of Chemistry, Chemical Engineering and Materials Science, Collaborative Innovation Center of Functionalized Probes for Chemical Imaging in Universities of Shandong, Key Laboratory of Molecular and Nano Probes, Ministry of Education, Shandong Provincial Key Laboratory of Clean Production of Fine Chemicals, Shandong Normal University, Jinan 250014, P. R. China.

E-mail: tangb@sdnu.edu.cn

Experimental Section:

Materials and Reagents. 1-Octadecene (ODE), Rare earth oxides Yttrium(III) chloride (YCl_3), Ytterbium(III) chloride ($YbCl_3$), Thulium(III) chloride ($TmCl_3$) and 3-(4,5-dimethyl-thiazol-2-yl)-2,5-diphenyltetrazolium bromide (MTT) were purchased from Sigma Chemical Company; Oleic acid (OA), (4-Carboxybutyl)triphenylphosphonium bromide, (3-aminopropyl)triethoxysilane (APTES), 1-ethyl-3-(3-dimethylaminopropyl) carbodiimide hydrochloride (EDC) and N-hydroxysuccinimide (NHS) were purchased from Alfa Aesar Chemical Ltd (tianjin, China); Tetrabutyl titanate (TBOT) was purchased from China National Pharmaceutical Group Corporation (Shanghai, China). Oleic acid and 1-Octadecene were of technical grade and the others were of analytical grade. All the chemicals were used without further purification. The human breast cancer cell line (MCF-7) was purchased from KeyGEN biotechnology Company (Nanjing, China).

Instruments. High resolution transmission electron microscopy (HRTEM) was carried out on a JEM-2100 electron microscope. Fluorescence spectra were obtained with FLS-920 Edinburgh Fluorescence Spectrometer with a Xenon lamp. Absorption spectra were measured on a pharmaspec UV-1700 UV-visible spectrophotometer (Shimadzu, Japan). All pH measurements were performed with a pH-3c digital pH-meter (Shanghai LeiCi Device Works, Shanghai, China) with a combined glass-calomel electrode. Absorbance was measured in a microplate reader

(Synergy 2, Biotek, USA) in the MTT assay. Confocal fluorescence imaging studies were performed with a TCS SP5 confocal laser scanning microscopy (Leica Co., Ltd. Germany) with an objective lens (×40, ×60). The sections were also observed through Nikon eclipse 80i microscope.

Synthesis of $NaYF_4$:20%Yb^{3+},0.1%Er^{3+},0.2%Tm^{3+} nanocrystals. The β-phase $NaYF_4$:Yb^{3+},Er^{3+},Tm^{3+} nanoparticles were synthesized using a solvothermal method with some modifications. Briefly, 1 mmol rare earth chlorides YCl_3, $YbCl_3$, $ErCl_3$ and $TmCl_3$ with a stoichiometric ratio of 79.7:20:0.1:0.2 were dispersed in 8 mL of oleic acid and 18 mL of 1-octadecene, and then the mixture was heated to 156 ^{o}C for half an hour under stir. After a homogeneous solution was formed, the mixture was cooled down to room temperature, then 10 mL of methanol solution containing sodium hydroxide (0.1 g, 2.5 mmol) and NH_4F (0.148 g, 4 mmol) was added dropwise. After stirring for 30 min, the temperature was raised to 100 ^{o}C to make the methanol volatilizing. Then the mixture was heated to 295 ^{o}C in an argon atmosphere for 90 min and cooled to room temperature. The precipitates were washed with methanol and cyclohexane (1:1, v/v) for times and redispered in 10 mL of cyclohexane.

Synthesis of UCNPs@TiO_2 core-shell nanoparticles. Before the synthesis, the oleic acid ligand on the surface of upconversion nanoparticles should be removed firstly. 0.5 mL of as-synthesized oleate-conjugated UCNPs was dispersed in a 10 mL of HCl solution (pH=2) under vigorous stirring for 4 h to make the carboxylate groups of the oleate ligand protonated (to yield oleic acid). After the reaction was completed, diethyl ether was added to the solution to remove the oleic acid by extraction. Then the oleate-free UCNPs in aqueous phase were obtained by centrifugation. The UCNPs@TiO_2 core/shell nanoparticles were synthesized via a kinetics-controlled method.[45] The OA-free upconversion nanoparticles were dispersed in 25 mL absolute ethanol and mixed with concentrated ammonia solution (75 μL, 28 wt%) under ultrasound for 15 min. Subsequently, 120 μL of tetrabutyl titanate (TBOT) was added dropwise in 5 min, and the reaction was performed for 24 h at 45 ^{o}C under continuous gentle stirring. The final products were separated under centrifugation, followed by washing with deionized water and ethanol for 3 times, respectively. After dried at 100

^{o}C, the UCNPs@TiO_2 was gained and redispersed in deionized water for further use.

Synthesis of UCNPs@TiO_2-Ce6-TAT. We synthesized UCNPs@TiO_2-NH_2 at first. 2 mg as-synthesized UCNPs@TiO_2 was dispersed in a mixture containing 10 mL of ethanol, 200 μL of deionized water under stirring for 15 min. And then 8 μL of APTES was added into the solution and the mixture was stirred for another 12 h to modify the amino groups on the surface of UCNPs@TiO_2. The resulting nanoparticles were collected and washed with ethanol and deionized water for several times, and finally dispersed in MES buffer (10 mM, pH=6.0). UCNPs@TiO_2-Ce6-TAT was obtained by coupling the carboxyl groups of Ce6 and TAT peptides with the amino groups on the surface to form the amido bonds. 10 μmol of EDC and 10 μmol of NHS were mixed with 2 μmol of Ce6 in MES buffer in the dark for 30 min to activate the carboxyl groups. 2 mg UCNPS@TiO_2-NH_2 was then added to the solution under stirring for 12 h. The precipitates were centrifuged and washed with PBS buffer (pH=7.4). 75 μmol of EDC and 75 μmol of NHS were mixed with 15 μmol of TAT peptides in PBS buffer to activate the carboxyl groups for 30 min to activate the carboxyl groups as well. Then the activated TAT peptides were added to UCNPs@TiO_2-Ce6 solution under stirring for 12 h to form final product UCNPs@TiO_2-Ce6-TAT. The prepared nanoparticles were washed with deionized water for several times and redispersed in water for further use.

Cell culture. MCF-7 cells were cultured in Dulbecco's modified Eagles medium (DMEM) with 10% fetal bovine serum and 100 U/ml 1% antibiotics penicillin/streptomycin and maintained at 37 °C in a 100% humidified atmosphere containing 5% CO_2. And MCF-7/Dox were cultured in RPMI-1640 with 10% fetal bovine serum, 100 U/ml 1% antibiotics penicillin/streptomycin and doxorubicin (1 μg/mL) and maintained at 37 °C in a 100% humidified atmosphere containing 5% CO_2.

Co-localization into nucleus. MCF-7 cells were first cultured in a confocal dish for 24 h. Then, the cells were treated with UCNPs@TiO_2-Ce6-TAT and UCNPs@TiO_2-Ce6 (0.1 mg/mL) in DMEM culture medium. After 4 h incubation, cells were washed several times with PBS buffer to remove the nanoparticles outside the cells. Then,

fresh DMEM culture medium was added and the cells were incubated for another 8h. Then, the cells were washed with PBS twice and stained with Hoechst 33342 for 15 min. Subsequently, the cells were observed using confocal laser scanning microscopy (CLSM) and confocal images were captured with 405 nm excitation for Hoechst 33342 (emission=430-480 nm) and 633 nm excitation for Ce6 (emission=650-700 nm). The liner fluorescence profile was quantified using Image-Pro Plus Imaging software.

Intracellular tracking UCNPs@TiO$_2$-Ce6-TAT. MCF-7 cells were cultured in a confocal dish for 24 h. Then, the cells were treated with UCNPs@TiO$_2$-Ce6-TAT (0.1 mg/mL) in DMEM culture medium and maintained at 37 °C in 5% CO_2. After incubation for 4 h, cells were then washed several times with PBS buffer to remove the excess nanoparticles outside the cells. Then, 2 mL fresh DMEM culture medium was added and the cells were further cultured for 4, 8, and 20 h. Subsequently, the cells were stained with Hoechst 33342 at 37 °C for 15 min. The cells were then rinsed with PBS and observed using CLSM. The confocal images were captured with 405 nm excitation for Hoechst 33342 and 633 nm excitation for Ce6.

***In vitro* therapeutic effects.** (1) MCF-7 cells were cultured in 96-well microtiter plates and incubated at 37 °C in 5% CO_2 for 24 h. UCNPs@TiO$_2$-Ce6-TAT (0.1 mg/mL) was incubated with the cells in DMEM culture medium for 12 h followed by washing the cells with PBS buffer to remove the nanoparticles that were not uptake into the cells. Then, MCF-7 cells were treated with irradiation of different laser powers and irradiation times (0.5, 1, 2 W·cm^{-2} and 1, 2, 3, 4, 5 min, respectively). The cells in the control group were without any treatment. Then, the cells were further cultured for 24 h. 150 μL MTT solution (0.5 mg/mL) was then added to each well. After incubation for 4 h, the MTT solution was removed, and 150 μL of DMSO was added to each well under slight shake in the dark. Considering the therapeutic effects and the lower phototoxicity to normal cells, the parameter of 1 W·cm^{-2} and 5 min was chosen in the following therapeutic application. (2) MCF-7 cells were cultured in 96-well microtiter plates and incubated at 37 °C in 5% CO_2 for 24 h. UCNPs@TiO$_2$-Ce6, UCNPs@TiO$_2$-TAT, and UCNPs@TiO$_2$-Ce6-TAT (0.1 mg/mL) were incubated with

the cells in DMEM culture medium for 12 h, respectively, followed by washing the cells with PBS buffer to remove the nanoparticles that were not uptake into the cells. Then, MCF-7 cells were treated with 980 nm laser irradiation (1 $W{\cdot}cm^{-2}$) for 5 min. The cells in the control group were without any treatment. Then, the cells were further cultured for 24 h. Then, 150 μL of MTT solution (0.5 mg/mL) was added to each well. After incubation for 4 h, the MTT solution was removed, and 150 μL of DMSO was added to the well under slight shake in the dark. (3) MCF-7 cells were cultured in 96-well microtiter plates and incubated at 37 °C in 5% CO_2 for 24 h. UCNPs@TiO_2-Ce6-TAT (0.1 mg/mL) without irradiation and only irradiation with 980 nm laser (1 $W{\cdot}cm^{-2}$, 5 min) were performed on two groups of cells, respectively. The cells in the control group were without any treatment. Then, the cells were further cultured for 24 h. Then all groups of cells were washed with PBS buffer to remove the nanoparticles that were not uptake into the cells. Afterward, 150 μL of MTT solution (0.5 mg/mL) was added to each well. After incubation for 4 h, the MTT solution was removed, and 150 μL of DMSO was added to the well under slight shake in the dark. The absorbance was measured at 490 nm with microplate reader.

***In vitro* DNA damage study.** DNA damage was detected using H2A.X Phosphorylation Assay Kit. Take MCF-7 cells for example. In brief, $2{\times}10^6$ MCF-7 cells were incubated with UCNPs@TiO_2-Ce6, UCNPs@TiO_2TAT, UCNPs@TiO_2-Ce6-TAT (0.1mg/mL) for 12 h, respectively, and treated with 980 nm laser irradiation for 5 min. Then each group of cells was trypsinized and centrifuged (1000 rpm, 3 min). The cells were resuspend with 1X fixation solution at a cell density of $2{\times}10^6$ per mL and incubated for 20 min in ice to fix the cells, followed by washed the cells using PBS to remove the fixative. The cells pellets were then gently resuspend in 1X permeabilization solution to a density of $2{\times}10^6$ cells per mL (50 μL per $1{\times}10^5$ cells). Subsequently, either FITC conjugated anti-phospho-histone H2A.X (Ser139) or the negative control FITC conjugated normal mouse IgG (3.5 μL/$1{\times}10^5$ cells) was added and the cells were incubated on ice for 20 min. Then add 1 mL of 1X wash solution aid in removing excess FITC labeled antibody. Cell images were acquired using the ImageStream^X multispectral imaging flow cytometer (Amnis Corporation) with 488

nm excitation for FITC (emission=500-560 nm). Cell images were analyzed using IDEAS® image analysis software (Amnis).

Animal tumor xenograft models. All animal experiments were carried out and accorded with the Principles of Laboratory Animal Care (People's Republic of China). Female nude mice (4-6 week old, ~20 g) were raised on normal conditions of 12 h light and dark cycles and given access to food and water *ad libitum*.

For xenografts established from cultured cells, MCF-7 cells or MCF-7/Dox cells were suspended via trypsinization and collected by centrifugation (1000 rpm, 3 min) and approximately 1×10^6 MCF-7 cells or MCF-7/Dox cells in 150 μL PBS were injected subcutaneously into right of the nude mice. The tumor volume (V) was calculated as $V=L\times W^2/2$ by measuring length (L) and width (W). The relative tumor volumes were calculated for each sample as V/V_0 (V_0 was the original tumor volume). The treatments were administrated only once when the tumor volume reach to about 150 mm^3.

***In vivo* antitumor efficacy via injection.** When the tumor volume reached to about 150 mm^3 the tumor-bearing mice were weighed and randomly divided into different groups ($n\geq 5$). The mice with two different xenograft tumors were subjected with different treatments: PBS (50 μL) only, UCNPs@TiO_2-Ce6 (50 μL 1.0 mg/ml) combined with laser irradiation, UCNPs@TiO_2-TAT (50 μL 1.0 mg/ml) combined with laser irradiation, UCNPs@TiO_2-Ce6-TAT (50 μL 1.0 mg/ml) combined with laser irradiation. Laser treatment was performed by irradiating the tumor region with 980 nm laser at a power of 1 $W\cdot cm^{-2}$ for 5 min at 12 h after intratumor injection. The tumor size and the body weight of each mouse were measured every two days within 14 days.

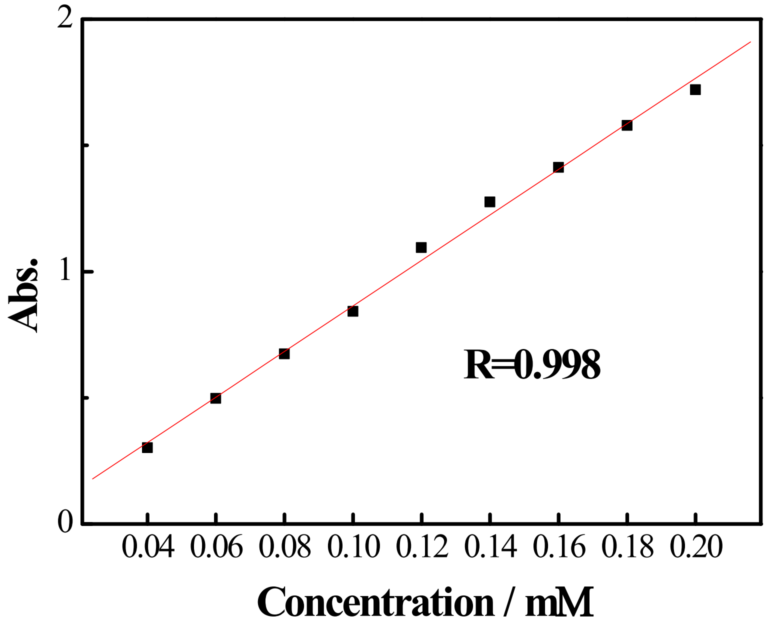

Figure S1. Standard linear calibration curve of Ce6.

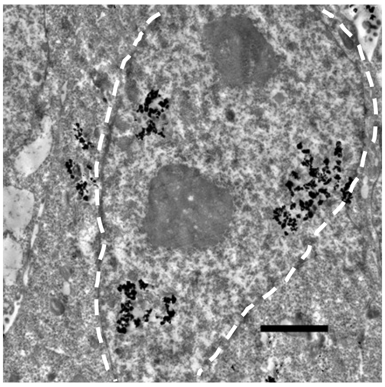

Figure S2. Bio-TEM images of MCF-7 cells loaded UCNPs@TiO_2-Ce6-TAT.

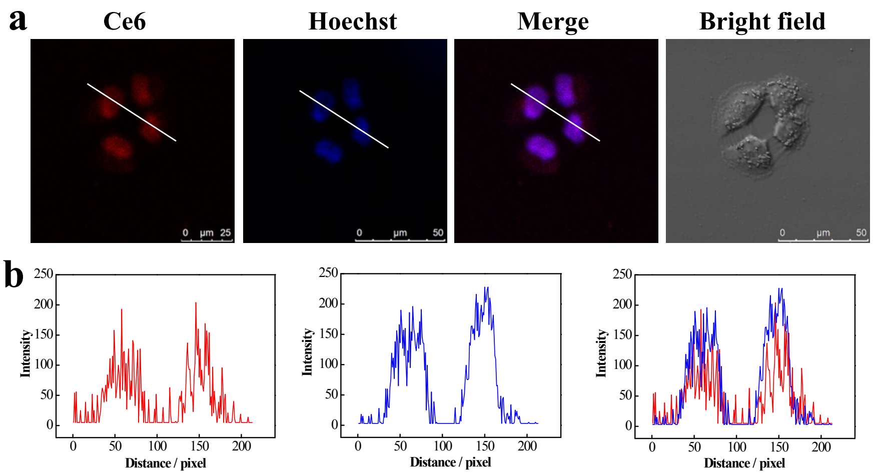

Figure S3. Nuclear targeting assay of MCF-7/Dox cells. (a) Co-localization images of the nuclear targeted dual-photosensitizer and the nuclei using confocal laser scanning microscopy (CLSM). MCF-7/Dox cells were incubated with UCNPs@TiO_2-Ce6-TAT for 12 h before measurement. Confocal images of nuclear targeted dual-photosensitizer (λ_{ex} =633 nm, λ_{em} =650-700 nm), Hoechst 33342 stained nuclei (λ_{ex}=405 nm, λ_{em}=430-480 nm) and the overlay channel. (b) The quantification of fluorescent intensity of the line scanning profiles in the corresponding confocal images in a.

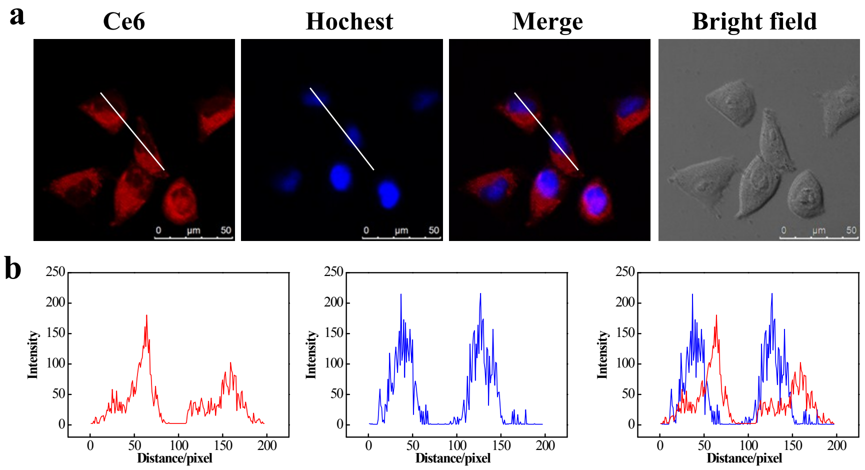

Figure S4. (a) Confocal images of MCF-7 cells loaded UCNPs@TiO2-Ce6 without TAT peptides. The nuclei were stained with hoechst 33342. (b) The quantification of fluorescent intensity of the line scanning profiles in the corresponding confocal images in a.

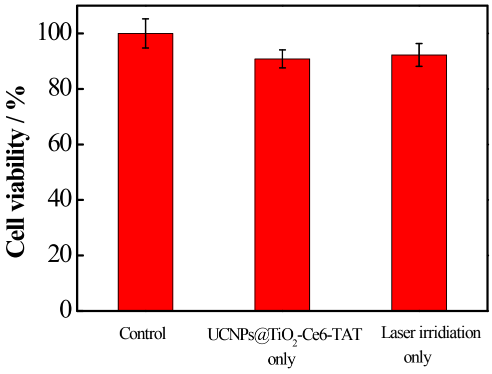

Figure S5. Cell viability. The viability of cells treated with UCNPs@TiO_2-Ce6-TAT only and laser irradiation for 5 min only.

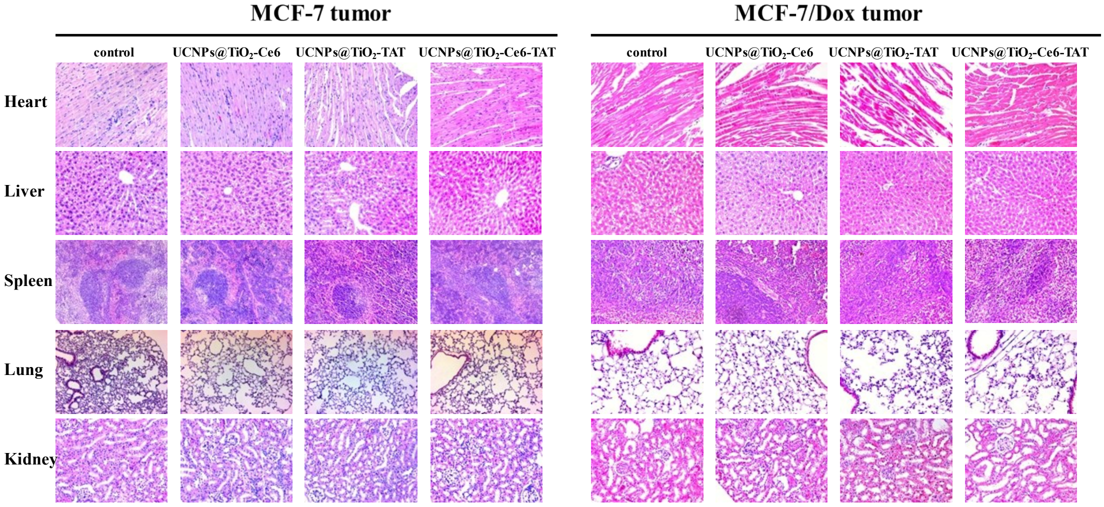

Figure S6. H&E staining images of five major organs (heart, liver, spleen, lung, and kidney) in MCF-7 tumor-bearing mice (left) and MCF-7/Dox tumor-bearing mice at 7 day after different treatment groups: control, UCNPs@TiO_2-Ce6, UCNPs@TiO_2-TAT, and UCNPs@TiO_2-Ce6-TAT with irradiation for 5 min at the tumor region. The power of the irradiation was 1 W·cm^{-2}. And no histopathological abnormalities were observed in each group.
